# Plastome Diversity and Phylogenomic Relationships in Asteraceae

**DOI:** 10.3390/plants10122699

**Published:** 2021-12-08

**Authors:** Joan Pere Pascual-Díaz, Sònia Garcia, Daniel Vitales

**Affiliations:** 1 Institut Botànic de Barcelona (IBB-CSIC), Passeig del Migdia s/n, 08038 Barcelona, Spain; joanpere.pascual@ibb.csic.es; 2 Laboratori de Botànica–Unitat Associada CSIC, Facultat de Farmàcia i Ciències de l’Alimentació, Universitat de Barcelona, Av. Joan XXIII 27-31, 08028 Barcelona, Spain

**Keywords:** chloroplast genome, Compositae, phylogenetic incongruence, plastid DNA, Senecioneae

## Abstract

Plastid genomes are in general highly conserved given their slow evolutionary rate, and thus large changes in their structure are unusual. However, when specific rearrangements are present, they are often phylogenetically informative. Asteraceae is a highly diverse family whose evolution is long driven by polyploidy (up to 48*x*) and hybridization, both processes usually complicating systematic inferences. In this study, we generated one of the most comprehensive plastome-based phylogenies of family Asteraceae, providing information about the structure, genetic diversity and repeat composition of these sequences. By comparing the whole-plastome sequences obtained, we confirmed the double inversion located in the long single-copy region, for most of the species analyzed (with the exception of basal tribes), a well-known feature for Asteraceae plastomes. We also showed that genome size, gene order and gene content are highly conserved along the family. However, species representative of the basal subfamily Barnadesioideae—as well as in the sister family Calyceraceae—lack the pseudogene *rps*19 located in one inverted repeat. The phylogenomic analysis conducted here, based on 63 protein-coding genes, 30 transfer RNA genes and 21 ribosomal RNA genes from 36 species of Asteraceae, were overall consistent with the general consensus for the family’s phylogeny while resolving the position of tribe Senecioneae and revealing some incongruences at tribe level between reconstructions based on nuclear and plastid DNA data.

## 1. Introduction

The sunflower family (Asteraceae or Compositae) is probably the most diversified of plants, with about 25,000–35,000 species, being distributed worldwide and accounting for ca. 10% of angiosperms [1,2]. The family contains many important crops (such as lettuce, sunflower or artichoke) and many ornamentals (such as marigolds or dahlias) but also many weeds (such as dandelion or some thistles) [1]. Two of the defining morphological traits of the family have been crucial for the evolutionary and ecological success of Asteraceae: the characteristic inflorescence in the capitulum, in which many tiny flowers (florets) are packed in a receptacle, and the cypsela, an indehiscent dry fruit derived from a compound inferior ovary, which usually has adaptations for an effective dispersal and to herbivory [3,4]. Asteraceae has long been the subject of cytological interest, and it is possibly the plant family for which more chromosome counts are available, with *x* = 9 as the most likely ancestral base number. Hybridization and polyploidy are particularly active in the family, with ploidy levels up to 48*x* [5], and indeed several whole-genome duplications (WGDs) [6], together with frequent hybridization phenomena [7,8], are linked to the massive diversification of Asteraceae, complicating, at the same time, its systematics. Other cytological features, such as the presence of an exceptional linked arrangement of ribosomal RNA genes in many of its species [9] add interest to the study of this family.

As already supported by early molecular phylogenetic studies [10], Asteraceae constitutes a well-defined family that originated in South America, its most closely related families being Calyceraceae (South American origin) and Goodeniaceae (Southwestern Australian origin) [11,12]. Resolving the systematic relationships within Asteraceae has been, however, a more challenging task. Since the first molecular-based approaches [13,14] to the major compilation and meta-tree analyses by [1], plastid DNA has been a preferred target of researchers interested in Asteraceae evolution. The combination of slowly evolving genic regions and the fast evolutionary rate of intergenic spacers has made plastid markers classical candidates for phylogenetic reconstruction at different taxonomic levels within the family [15]. One of the latest and most comprehensive evolutionary reconstructions at the family level was based on DNA sequences of 11 plastid genes [16]. More recently, the backbone of Asteraceae phylogeny was successfully resolved using “targeted sequence capture” data from 935 nuclear loci [17]. However, some phylogenetic uncertainties, as well as a few incongruences between nuclear and plastid inferences, still remain.

From the structural point of view, plastomes in angiosperms are 120–160 kb long and have a quadripartite structure with two single-copy regions (LSC, long, and SSC, short single copy) separated by two inverted repeats (IRA and IRB) [18]. Each plastid genome of Asteraceae investigated to date is around 150 kb long and contains ca. 80 protein-coding genes, four ribosomal RNAs (rRNAs) and 30 transfer RNAs (tRNAs) in the expected quadripartite organization [19]. Large changes in plastid DNA structure across land plants are unusual, but some families such as Geraniaceae, Fabaceae and Ericaceae, do show several plastome rearrangements (e.g., expansions, contractions, inversions or losses of an IR; [20]), in many cases reported as phylogenetically informative. In Asteraceae, all plastomes—except those from Barnadesioideae, a small basal subfamily with roughly 100 species—share a distinctive structural feature: a double inversion in the plastid DNA [14]. Both inversions are located in the LSC region, with the larger inversion (~22.8 kb long) containing the second one (~3.3 kb long). These inversions have been confirmed using both Sanger and next-generation sequencing (NGS), the latter from a few species of the family (e.g., *Artemisia capillaris* [21]). However, further studies including a larger representation of species could provide new insights into the structural variability of Asteraceae plastomes.

The advent of NGS technologies, as well as their continuously descending costs, has enabled the massive generation of genomic data for multiple species. In plants, whole-genome sequencing (WGS) datasets frequently include plastid DNA data. The development of de novo assembly bioinformatics tools such as NOVOPlasty [22] or SOAPdenovo2 [23], free and relatively easy to work with, has further facilitated the reconstruction of plastomes. Following those approaches, the plastid genomes of some species from Asteraceae have already been sequenced and published, now being stored in public repositories. However, the available genomic data represents a scattered and uneven taxonomic sampling, and thus more data are needed to analyze the diversity of plastomes within the family. In this study, we combined (i) seven previously published plastome reconstructions, (ii) seventeen new plastome assemblies obtained from WGS raw data stored in repositories and (iii) twelve new plastomes assembled from Illumina sequences specifically generated for this work. This strategy allowed us to build a comprehensive dataset with the whole plastid genome of 36 species representing the most important subfamilies and tribes of Asteraceae. We used these data to thoroughly characterize the plastome variability of the family. We also inferred the most complete plastid phylogenomic reconstruction carried out in Asteraceae, comparing our results to previous phylogenetic and phylogenomic studies on the family.

## 2. Results

### 2.1. Plastome Reconstruction in Asteraceae

The mixed sampling strategy, combining Illumina sequences generated for this study and raw genomic data obtained from public repositories, resulted in 30 new plastomes for Asteraceae, completely reconstructed and circularized. All these plastomes have the standard structure typically found in angiosperms, comprising two copies of the IR region (24,126 to 25,245 bp), separated by the LSC (82,297 to 85,288 bp) and the SSC (17,859 to 18,786 bp) regions (Table 1). Plastid lengths were similar in all species analyzed, *Achillea millefolium* being the species with the smallest plastid genome (149,113 bp) and *Melampodium linearilobum* the biggest one (153,872 bp). The GC content of the assembled plastomes ranged between 37% and 38.1%. After all quality controls, the read coverage per nucleotide ranged between 10 and 6021, and the percentage of Ns in whole-plastome reconstructions ranged between 0.00% and 3.80% (Table 1).

Asteraceae plastomes analyzed in this study encode 80 CDS, 30 tRNAs and 4 rRNAs, with an overall of 114 genes (Table S1). Two canonical CDSs were annotated as putative pseudogenes (Ψ) based on their altered structure and significantly higher nucleotide diversity: *ycf*1 (IR_B_) and *rpl*19 (IR_B_). Plastomes analyzed include 18 intron-containing genes, out of which 16 contain one intron (*trn*K-UUU, *rps*16, *rpo*C1, *atp*F, *trn*G-UCC, *trn*L-UAA, *trn*V-UAC, *rps*12, *pet*B, *pet*D, *rpl*16, *ndh*A, *trn*A-UGC, *trn*I-GAU, *ndh*B, *rpl*2), while two contain two introns (*clp*P and *ycf*3).

### 2.2. Phylogenetic Analysis

**Figure 1:**
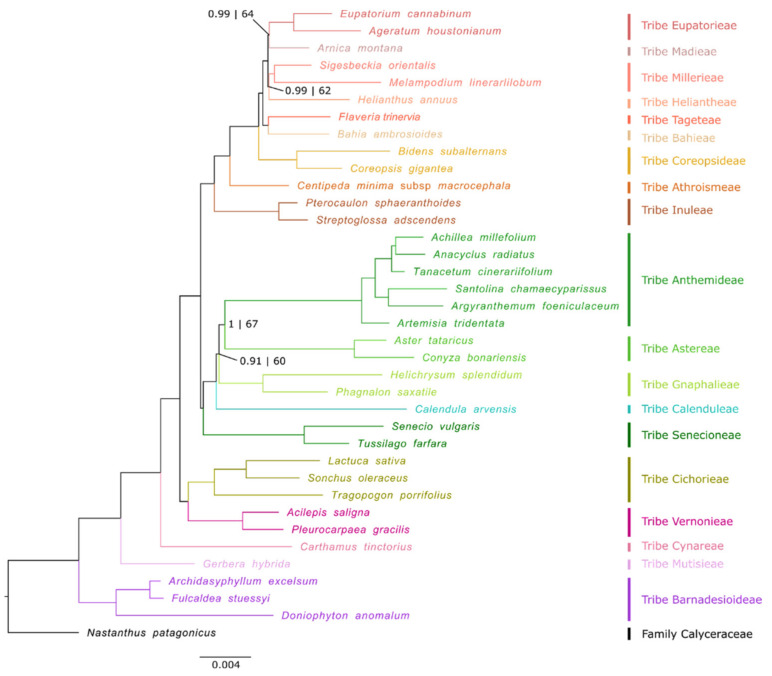
Phylogenomic tree of 37 species derived from 77,259 bp of the plastid coding DNA sequences (CDSs), transfer RNAs (tRNAs) and ribosomal RNAs (rRNAs), using maximum likelihood (ML) and Bayesian inference (BI) approaches. Nodes without numbers are considered nodes with maximum support for both ML and BI approaches. Nodes with numbers are spots with low support in at least one phylogenetic inference approach (posterior probability/bootstrap). Low support is defined as: posterior probability < 1; bootstrap < 70.

The resulting tree topology from Bayesian and ML inferences is shown in Figure 1. BI and ML trees were identical in topology (Figure S1), both phylogenetic approaches being highly resolved but BI showing stronger support in a few branches. In addition, phylogenetic inferences drawn with different combinations of CDSs, tRNA and rRNA and species datasets (Figure S1) to validate the topology of the tree were congruent with the main topology result. The phylogenetic positions of all early-divergent clades of the family Asteraceae (i.e., subfamilies Barnadesioideae, Mutisioideae and Carduoideae) are well-supported (pp = 1, BS = 100, in all cases). The monophyly of subfamily Cichorioideae is also well-supported, being placed as the sister group of the subfamily Asteroideae (pp = 1, BS = 100). The subfamily Asteroideae is divided into two major clades, one representing the Helianthodae supertribe and another constituted by supertribes Asterodae and Senecionodae [16,24], here grouped in a fully supported clade. In the Helianthodae supertribe, the node grouping tribes Heliantheae and Millerieae only shows high support in the BI tree (pp = 0.99, BS = 62), the same pattern occurring in the group constituted by tribes Eupatorieae and Madieae (pp = 0.99, BS = 64). In the Asterodae + Senecionodae clade, there are only two nodes that do not show the highest support according to both phylogenetic approaches: the split between tribe Gnaphalieae and tribes Anthemideae and Astereae (pp = 0.91, BS = 60) and the branch grouping tribes Anthemideae and Astereae (pp = 1, BS = 67).

### 2.3. Structural Comparison of Plastomes

**Figure 2:**
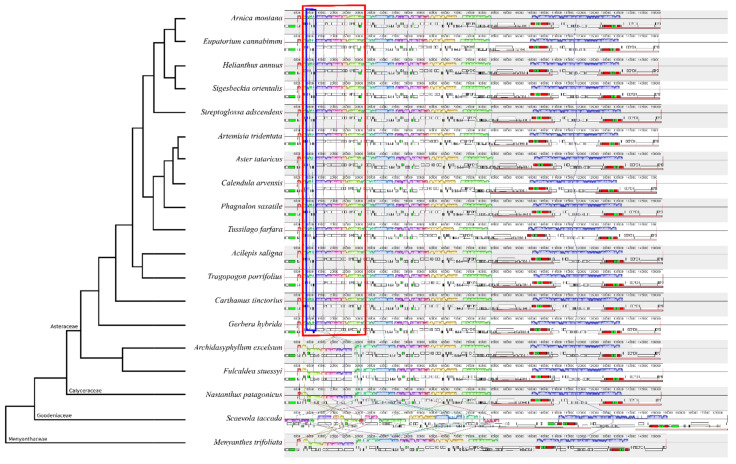
Mauve alignment of a subset of 19 plastid genomes of species from families Asteraceae, Calyceraceae, Goodeniaceae and Menyanthaceae, sorted phylogenetically. Within each of the alignments, local collinear blocks are represented by blocks of the same color and connected by lines. All Asteraceae except species from subfamily Barnadesioideae (*Archidasyphyllum excelsum* and *Fulcadea stuessy*) share a double inversion (the larger indicated in red, and the smaller, nested within the previous, indicated in blue).

All Asteraceae plastomes, together with those of outgroups *Nastanthus patagonicus* (Calyceraceae), *Scaevola taccada* (Goodeniaceae) and *Menyanthes trifoliata* (Menyanthaceae), were used to perform a comparative analysis of plastid DNA structure across the MGCA clade and within family Asteraceae. The results obtained from this analysis (Figure 2 and Figure S2) indicate that most Asteraceae plastomes exhibit a high level of sequence similarity and structural conservation. The analysis confirms the existence of a rearrangement in the LSC consisting of a double inversion: one large inversion of ~22.8 kb and one small inversion of ~3.3 kb nested within the large one. This rearrangement is present in all species of subfamilies Mutisioideae, Carduoideae, Cichorioideae and Asteroideae, while it does not appear in subfamily Barnadesioideae nor in the sister family Calyceraceae (Figure 2). Family Menyanthaceae has the same plastome structure as Calyceraceae and the members of the tribe Barnadeiosideae. However, the plastome of the species representing Goodeniaceae, the sister family to the clade constituted by Calyceraceae and Asteraceae, is full of rearrangements—not only in the LSC region but also in the SSC and in both IRs—and thus its LSC structure could not be compared with the other families.

**Figure 3:**
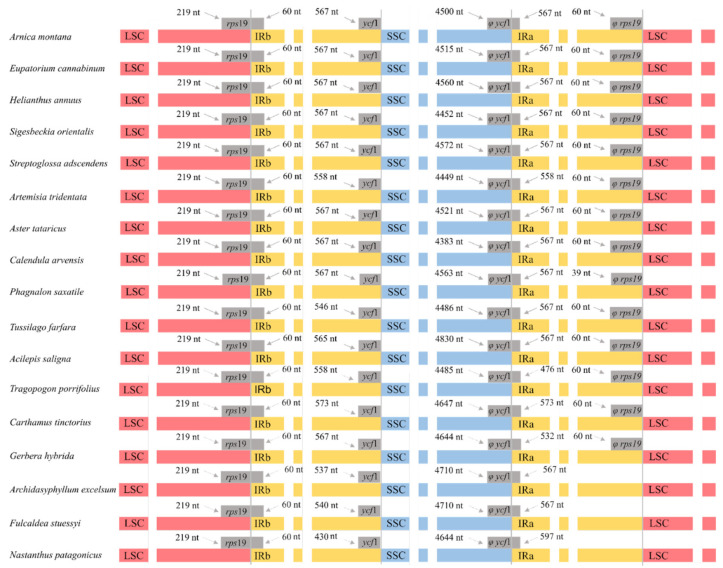
Comparison of the boundaries of the LSC, SSC and IR regions of a subset of 17 species from families Asteraceae and Calyceraceae (*Nastanthus patagonicus*). Genes suffixed with a phi (φ) are potential pseudogenes.

By visualizing the expansions and contractions of the boundaries of the IR regions (Figure 3), we also found an additional structural difference in the plastome shown by the family Calyceraceae and the subfamily Barnadesioideae as compared to the plastomes of the remaining Asteraceae. Most Asteraceae species harbor the same genes in the boundaries between IR regions and single-copy regions (LSC/IR_A_: *rps*19; IR_A_/SSC: *ycf*1; SSC/IR_B_: ^Ψ^*ycf*1; IR_B_/LSC: ^Ψ^*rps*19). However, species from subfamily Barnadesioideae and the Calyceraceae *Nastanthus patagonicus* lack the ^Ψ^*rps*19 pseudogene localized in the boundary between the IR_B_ and the LSC (Figure 3 and Figure S3).

### 2.4. Characterization of Sequence Divergence, Repeats and SSRs

**Figure 4:**
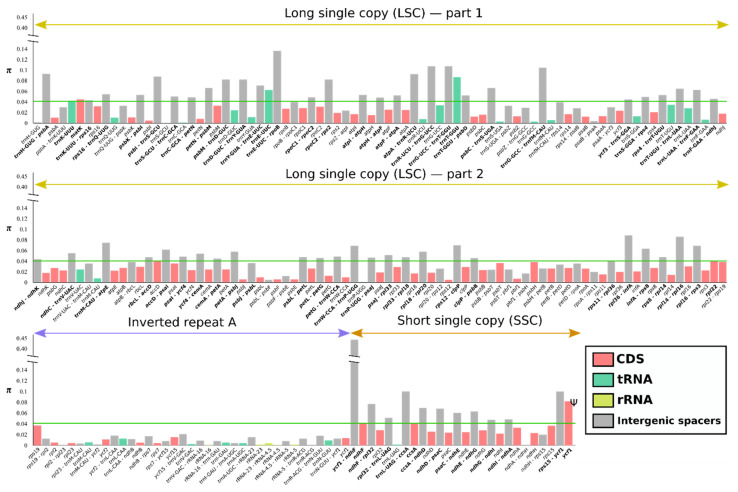
Nucleotide diversity (π) through 36 species of family Asteraceae. Different coding and non-coding regions (CDS, tRNA, rRNA, IGS) are indicated by colors. The nucleotide diversity threshold (0.04) is indicated by the green line.

We compared coding genes, tRNA, rRNA and intergenic spacers across all Asteraceae species examined to find nucleotide divergence hotspots. For the 225 regions analyzed (29 tRNA, 83 CDS, four rRNA and 109 intergenic spacers) the π value within Asteraceae ranged from 0 (*ndh*A-*ndh*H, *trn*R-ACG, *rpl*2-*rpl*23) to 0.43814 (*ycf*1-*ndh*F) (Figure 4). Considering the whole family, most of the intergenic spacers could be regarded as highly divergent regions, surpassing the threshold of π = 0.05, with the exception of the inverted repeat, where any region outweighs the threshold value.

For the entire plastid genomes, the IR regions (IR_A_ π = 0.00768, IR_B_ π = 0.00791) were more conserved than both the LSC (π = 0.03611) and the SSC (π = 0.05974). Both IRs recovered the same nucleotide diversity for almost all genes, being mirror images one of the other. Regarding the different genomic regions analyzed in this study, the tRNA and the rRNA were the regions with lower nucleotide diversity (tRNA π = 0.01558, rRNA π = 0.00109), followed by the CDS (π = 0.02238) and the intergenic spacers (π = 0.04971).

We classified sequence dispersed repeat motifs into four categories: forward, reverse, palindromic, complement reverse and tandem repeats. In general, palindromic and reverse repeats were the most common, and a low proportion of complement reverse repeats was only identified in *Aster tataricus* (1), *Bidens subalternans* (2), *Conyza bonariensis* (1), *Helianthus annuus* (4)*, Helichrysum splendidum* (2), *Pleurocarpaea gracilis* (3) and *Archidasyphyllum excelsum* (1) (Figure S4). Tandem repeats were distributed among all the species, from 18 (*Arnica montana*) to 54 (*Conyza bonariensis*) (Figure S5).

Microsatellites or SSRs were detected in every species analyzed, and Asteraceae plastid genomes contained from 124 (*Argyranthemum foeniculaceum*) to 234 (*Conyza bonariensis*) microsatellites (Figure S5). All plastomes analyzed had four types of SSRs (mono-, di-, tri- and tetranucleotides), and a few also contained penta- and hexanucleotides (Table S3). Most microsatellites were mononucleotides in all Asteraceae and Calyceraceae species (on average, mono-: 66.77%, di-: 24.98%, tri-: 2.81%, tetra-: 4.40%, penta-: 0.79%, hexanucleotides: 0.25%). Almost all mononucleotides were highly AT-rich (97.12%), and AT/TA repeats were also the most common among dinucleotide microsatellites (55.73%).

## 3. Discussion

In this study, we analyzed 36 complete plastid genomes from species of a single family, 30 of which were de novo assembled for the first time. This extensive sampling helped us understand the structural diversity of Asteraceae plastid genomes in a phylogenetic context, covering the most relevant tribes of the family. Our work also constitutes the most complete phylogenomic approach based on whole plastid genomes performed in Asteraceae, complementing the recent evolutionary studies of the family based on nuclear and plastid phylogenomic data.

### 3.1. Structural and Nucleotide Diversity of Asteraceae Plastomes

The plastid genomes of the species here studied present the typical quadripartite structure, and they are in the genome size range for land plants (Table 1, Figure 1) [25]. The length of plastomes included in our analyses showed a maximum variability of 4759 bp, which is congruent with the length differences found in previous Asteraceae plastomes published in GenBank. Usually, plastid genomes exist in two different structural haplotypes within an individual, differing in the orientation of the LSC or SSC [26]. We only detected one such structural haplotype in all the plastid genomes assembled in this study (Figure 2), most likely as an artifactual consequence of the plastome reconstruction method based on short reads; possibly, the use of long reads would have revealed the existence of both haplotypes [27]. Previous comparative analyses of plastomes in Asteraceae show similar results regarding structure, genome size and haplotype found [27,28,29]. While the structure of all chloroplast genomes analyzed was highly conserved, when early-divergent Asteraceae (i.e., subfamily Barnadesioideae) were compared with the rest of the family, two rearrangements were found in the LSC region (Figure 2): a large inversion of ~22.8 kb and a smaller ~3.3 kb inversion nested within it. This double inversion is localized in all major clades of Asteraceae, except in the early-diverging subfamily Barnadesioideae. The phylogenetic distribution of these rearrangements is consistent with previous results based on restriction endonuclease digestions, PCR and Sanger sequencing [14,30], which first detected these inversions and estimated that they originated during the late Eocene. In our study, we report for the first time that those Asteraceae species showing the double inversion in the LSC region (i.e., all of them except those belonging to Barnadesioideae) also present a pseudogenized *rps*19 at the end of the IR_B_. In contrast, Barnadesioideae subfamily as well as Calyceraceae family members show an alternative structure, lacking the pseudogenized *rps*19 (Figure 3) in one of the inverted repeats. Recent studies of plastome diversity show that the absence/presence of this pseudogene is scattered across angiosperms [31,32,33]. Our results suggest that this structural plastome feature (i.e., the presence of the pseudogenized *rps*19) might show a phylogenetic signal in Asteraceae, putatively co-occurring (or happening over a very short time span) with the double inversion event. However, this result must be taken with caution as [34] reported absence of the *rps*19 pseudogene in the Asteraceae species *Artemisia annua* while showing its presence in all the other Asteraceae and *Artemisia* species there analyzed.

The representative species from family Menyanthaceae (i.e., the sister family of Goodeniaceae, Calyceraceae and Asteraceae) seems to have a plastid genome structure similar to that found in Calyceraceae and in Barnadesioideae species. However, the representative of family Goodeniaceae included in the study shows a plastome structure completely altered by rearrangements as compared to the rest of the families within the MGCA clade. As recently reported by [35], plastomes from Goodeniaceae have many intergenic regions with a low GC proportion and many repeats, which may cause technical problems at the assembly level, possibly leading to an artefactual structure. Further work using new approaches of long-read sequencing (e.g., Oxford Nanopore or PacBio technologies) could help to correctly assemble and explore the sequence of Goodeniaceae plastid genomes [36].

At the level of genetic diversity, we found that intergenic spacers of Asteraceae have more than twice the nucleotide diversity of tRNA, rRNA and CDS, most likely because intergenic regions are not subject to selection constraints, allowing higher sequence divergence [37]. Regarding genic regions, tRNA and rRNA are more conserved than CDS, possibly due to the housekeeping function of the former two. There are also different levels of sequence conservativeness depending on the region, IR sequences being less variable than those of SSC and LSC, as reported previously [38,39,40]. The main reason could be, again, that IRs are harboring important housekeeping genes such as structural ribosomal RNA genes (*rrn*4.5, *rrn*23 and *rrn*16), highly conserved even in organisms with short IRs that possess only rRNA genes and few intergenic spacers, such as in some algae [41].

Due to the combination of fast- and slow-evolving regions, uniparental inheritance and ease of amplification, plastid DNA markers are among the preferred targets of many phylogenetic and phylogeographic studies. Based on their relatively high nucleotide divergence, we identified several genic regions that could be used as molecular markers in studies of Asteraceae species beyond the well-known CDS *mat*K [42] and *ndh*F [43], such as the CDS *rpl*22, *acc*D, *ccs*A and *ycf*1 and the tRNAs *trn*K-UUU, *trn*E-UUC and *trn*T-GGU.

Repetitive sequences play an important role in plastome rearrangements and could be useful to understand the evolution of plant species and sequence divergence [44]. The two main repeated motifs in plastomes are microsatellites and dispersed repeats [45]. The microsatellite data reported in our study can be useful as potential markers for evolutionary and genetic population analyses of Asteraceae [46,47]. Our results suggest that there are no relevant differences in the proportion of tandem repeats between tribes, and microsatellites are also in the expected range found in other studies, such as [45] (91 to 94 microsatellites in *Chaenomeles*), ref. [39] (172 microsatellites in *Hagenia*) and [48] (116 microsatellites in *Rubus*).

### 3.2. Asteraceae Plyogenomics Based on Plastid DNA

The availability of NGS data for an increasing number of species has hugely contributed to our understanding of the evolutionary relationships among organisms. Including data of 36 species from 19 tribes and 5 subfamilies of Asteraceae, this study represents the most comprehensive phylogenomic reconstruction using whole-plastome data obtained to date in this family (114 loci, 70,000 nt). Previous phylogenetic works had also approached the evolution of this huge and complex family, among the most relevant: (1) a meta-tree analysis combining results at lower taxonomic levels from several research works, based on 10 plastid DNA and one nuclear DNA marker (ITS) [1] and representing all tribes and subfamilies; (2) a phylogenetic inference based on 12 plastid markers representing 13 subfamilies and 40 tribes (17,319 nt) [16]; (3) an exhaustive phylogenomic study based on target sequence capture of 763 nuclear loci representing 13 subfamilies and 45 tribes (269,585 nt) [17]; and (4) a phylotranscriptomic analysis with data from 243 species (13 subfamilies and 41 tribes) within the family [49].

Our phylogenomic reconstruction based on plastome data showed overall consistency with the backbone tree topologies obtained in those previous approaches. However, unlike in the inferences by [1,16,17], we found a differential topology with strict support in basal nodes of subfamily Asteroideae, which could have important consequences for the taxonomy below the subfamily level. Study [1] placed tribe Senecioneae—the largest of the family, with ca. 150 genera and 3000 species—in a polytomy with the clade formed by the Inuleae + Athroismeae + Heliantheae Alliance (i.e., supertribe Helianthodae) and the clade containing Calenduleae + Gnaphalieae + Astereae + Anthemideae (i.e., Asterodae supertribe). Study [16] had previously found Senecioneae (there presented as supertribe Senecionodae) sister of Helianthodae and Asterodae supertribes, but the position lacked statistical support. Study [17] improved the resolution of important basal nodes in Asteroideae, merging Senecioneae with Anthemideae, Astereae, Gnaphalieae and Calenduleae (i.e., supertribe Asterodae). However, these authors found incongruences in the relationships of these five tribes among the trees they generated using different phylogenetic methods. While ML and BI yielded Calenduleae as an early-diverging group of the remaining tribes, the pseudocoalescence analysis (ASTRAL) resulted in high support for a different topology, with Senecioneae as the sister tribe to a clade of the four remaining Asterodae tribes. The same position of Senecioneae—despite not showing full statistical support—was obtained in the reconstruction of [49], also indicating that supertribe Senecionodae would not be monophyletic. Despite the limited sampling, our work provided full resolution and congruence among phylogenetic analyses for those basal nodes in subfamily Asteroideae, supporting the reconstructions obtained by [17] using the ASTRAL approach and by [49] using transcriptomic data, i.e., Senecioneae as a sister tribe to the clade constituted by Anthemideae, Astereae, Gnaphalieae and Calenduleae (i.e., supertribe Asterodae). Therefore, results based on plastome data—congruently with the last phylogenomic reconstructions based on nuclear data—suggest that the supertribe system for the subfamily Asteroideae early proposed by [24] could be recovered, merging tribe Senecioneae with the typical supertribe Asterodae.

As already obtained in previous analyses based on plastid DNA (e.g., [17]), our results support the monophyly of Cichorioideae, while recent phylogenomic approaches based on nuclear data reconstructed this subfamily as paraphyletic (e.g., [16]). According to [49], the different circumscription of Cichorioideae between the phylogenies using nuclear or chloroplast data could be explained by a potential hybridization during the evolution of Cichorieae. Our phylogenetic inference also differs from previous systematic reconstructions of the family based on nuclear and plastid markers in the relationships among the tribes of the Heliantheae alliance. As already reported by other phylogenetic studies mainly based on plastid DNA markers, such as [1,16], we found the important tribe Coreopsidae (550 spp.) in an early-diverging position within the Heliantheae alliance (Figure 1). In contrast, in the phylogenomic reconstructions based on nuclear data by [17] or [49], Coreopsidae is placed in a much-derived position as a sister tribe of Heliantheae s.s. There are other incongruences between plastid and nuclear genomes regarding the position of some tribes in the Heliantheae alliance. According to our results, as well as to other studies based on plastid DNA data (e.g., [16]), tribe Tageteae was sister to tribe Bahieae. In contrast, Tageteae was placed either as sister to Millerieae [17] or as sister to the weakly supported clade constituted by Madieae, Chaenactideae, Bahieae, Perityleae and Eupatorieae [49] in phylogenomic reconstructions based on nuclear DNA data. These incongruences between plastid and nuclear genomes have been explained by potential hybridization events involving plastid capture [16,49]. However, considering that the Heliantheae alliance is thought to be one of Asteraceae groups experiencing faster radiation [17], incomplete lineage sorting could also be here a possible source of phylogenetic incongruence [39].

## 4. Materials and Methods

### 4.1. Taxon Sampling

To obtain a representation as even as possible along the family Asteraceae, a mixed strategy of data gathering from online genome repositories and de novo sequencing was followed (Table 1). Information on the origin of plant material is shown in Table 2. First, the complete plastomes of *Archidasphyllum excelsum* [50], *Aster tataricus* [51], *Carthamus tinctorius* (GenBank: NC030783), *Conyza bonariensis* [52], *Doniophyton anomalum* (GenBank: MH899017), *Helianthus annuus* [53] and *Lactuca sativa* [54] were downloaded from GenBank. In addition, new assemblies were constructed using short-read archives (SRAs) from WGS projects corresponding to 19 species, downloaded from the European Bioinformatics Institute (EMBL-EBI). Additionally, twelve plastomes were assembled from Illumina sequences newly generated for this study. As outgroup taxa, we assembled the plastome of *Nastanthus patagonicus* (Calyceraceae) and downloaded from GenBank the complete plastomes of *Scaevola taccada* (Goodeniaceae, GenBank: NC040933) and *Menyanthes trifoliata* (Menyanthaceae, GenBank: MH201540), these three families constituting—together with Asteraceae—the “MGCA (Menyanthaceae + Goodeniaceae + Calyceraceae + Asteraceae) clade” (APG IV, 2016).

### 4.2. DNA Preparation and Sequencing

The total DNA of 13 species (Table 1) was isolated from dried leaf material using either a modified CTAB protocol [55] or the E.Z.N.A^®^ Plant DNA Kit (Omega Bio-tek, Inc., Norcross, GA, USA), depending on the quality and/or sufficient amount of material. Herbarium vouchers of the specimens are deposited at the Institut Botànic de Barcelona (IBB, CSIC-Ajuntament de Barcelona). The quality of each extraction was checked by spectrophotometry with NanoDrop 1000 (PeqLab, Erlangen, Germany) and the DNA concentration by fluorometry with Qubit Fluorometric Quantification (Thermo Fisher Scientific, Waltham, MA, USA). De novo random sequencing of whole DNA was performed by Beijing Genomics Institute (BGI; Shenzhen, China), employing an Illumina HiSeq X10 (Illumina, San Diego, CA, USA) platform, generating around 10 million paired-end reads (150 nt long) from ~500 bp insert size fragment libraries (Supplementary Table S1).

### 4.3. Genome Assembly and Annotation

The quality of all raw sequencing data—13 species sequenced for this study and 17 obtained from SRAs—was assessed by FastQC version 0.11.9 (http://www.bioinformatics.babraham.ac.uk/projects/fastqc/, accessed on 2 November 2020). Plastid genome reconstruction was performed with a mixed strategy combining de novo reconstruction of all plastomes and mapping assemblies of reads. De novo reconstruction of plastid sequences of 31 species was performed through the NOVOPlasty pipeline version 2.6.3 [22], using the raw whole dataset of Illumina reads, previously trimming the adapters, as recommended by the authors. After this de novo assembly, all raw data were additionally filtered based on the following rules: (i) adapter trimming; (ii) quality control: each read has >90% of bases with a quality cut-off value of >20. These filtering steps were carried out using CLC Genomics Workbench 10.0.1 (CLC-BIO, Aarhus, Denmark). These high-quality reads were then mapped to the circular reconstructions obtained with NOVOPlasty using Geneious version 2020.1.1 (Biomatters, Auckland, New Zealand) with the default mapping parameters, obtaining a consensus sequence for each species. All bases with <10 coverage were replaced by Ns.

Each plastome consensus was annotated using the software GeSeq [56] included in the platform MPI-MP CHLOROBOX (https://chlorobox.mpimp-golm.mpg.de/, accessed 10 December 2020), selecting the options to perform tRNAscan-SE version 2.0.3 and BLAT, using the reference sequences that are more phylogenetically proximal available in NCBI Ref Seq (https://www.ncbi.nlm.nih.gov/refseq/, accessed on 10 December 2020). Subsequently, all automatically annotated consensuses were checked manually using Geneious. The annotated plastid genomes were submitted to GenBank.

### 4.4. Plastome Phylogenetic Analyses

To estimate the phylogenetic relationships in Asteraceae based on plastome data, 36 species of this family were analyzed, together with one species of the sister family Calyceraceae (*Nastanthus patagonicus*) as outgroup, discarding species from families Goodeniaceae and Menyanthaceae to avoid amiss alignments. Both maximum likelihood (ML) and Bayesian inference (BI) analyses were performed on a dataset of 37 plastome sequences including a single IR (i.e., removing one of the IR copies to avoid redundant information), the SSC and LSC regions. All genes present in the dataset were extracted separately and then concatenated discerning between CDS, tRNA and rRNA; the non-coding regions were excluded from the analysis to be sure that all nucleotides were aligned with their homologous. The concatenated matrix was aligned using MAFFT version 7 [57] and then manually checked with Geneious, resulting in a dataset of 77,259 nucleotide sites. The aligned dataset was partitioned by separating the coding genes from the tRNA and rRNA, as well as by categorizing the nucleotides in each CDS based on the position they occupy in a codon (first, second or third). For the BI, the program MrBayes version 3.2.6 [58] included in the web-server CIPRES [59] (https://www.phylo.org/, accessed on 4 May 2021) was used to run two independent Markov chains Monte Carlo (MCMC) for 5,000,000 generations, with tree sampling every 1000 generations. The average standard deviation was confirmed to be less than 0.01, and the potential scale reduction factor was near 1.0 for all parameters. For the ML inference, the program RAxML version 8.2.10 [60] was used with 1000 bootstrap replicates and other parameters using the default settings. For both BI and ML approaches, PartitionFinder2 [61] was used to select the best evolutionary model for each concatenated region, chosen by selecting the scheme with the lowest AICc score. For the BI, the best partition scheme and substitution model was fitted for each region analyzed (Table S2). Regarding ML inference, RAxML allows for only a single evolutionary model in partitioned analyses, which was selected according to PartitionFinder2 results (i.e., GTRGAMMA). For both inferences, the first 25% of the trees were discarded as “burn-in”, and the posterior probabilities/bootstraps were estimated constructing the 50% majority-rule consensus tree.

To validate the topology obtained with the previous phylogenetic analysis and the support values obtained within the family Asteraceae, additional phylogenetic analyses using datasets with different combinations of genes and non-coding regions were performed. These subsets were: (i) CDS + tRNA + rRNA, (ii) CDS and (iii) the whole-plastid-genome sequences (Figure S1). Only the BI approach was used to perform the phylogenetic analysis for these datasets, employing the same parameter selection options mentioned above (Table S2).

### 4.5. Plastome Diversity Analyses

The complete plastomes of the Asteraceae analyzed in this study were used to estimate the structural and nucleotide diversity of plastid DNA within the family. Plastome rearrangements were checked among Asteraceae and the phylogenetically close families Calyceraceae, Goodeniaceae and Menyanthaceae (i.e., the “MGCA clade”; APG IV, 2016) to explore if the double inversion in the plastid DNA—as well as other possible structural changes—are apomorphies only found in Asteraceae or if these rearrangements are also present in close families. Structural changes across plastid genomes of Asteraceae and proximal families were analyzed via whole-genome alignment in Mauve version 2.4.0 [62], with the Mauve algorithm using default parameters. The expansion and contraction of the inverted repeat (IR) boundaries were also explored in order to check if these regions show differential patterns in length and gene annotations across species.

Previously to all analyses performed, the dataset containing all plastomes was aligned using MAFFT version 7 aligner [57] and then manually adjusted using Geneious. For the screening of the genetic variability between species, the nucleotide diversity (π) was estimated using DNAsp version 6 [63] for all coding DNA sequences (CDS), transfer RNA (tRNA), ribosomal RNA (rRNA) and intergenic spacers found in LSC, SSC and a single IR region.

To characterize repeat sequences in the plastid genomes, we used REPuter [64], with Hamming distance set at 3 and repeat range size from 30 to 90 bp, considering four types of repeats: forward, reverse, palindromic and complement sequences [65]. Tandem repeats were analyzed using the Tandem Repeats Finder [66] with default parameters. Short-sequence repeats (SSRs) were identified using the MISA microsatellite finder [67], with the following thresholds: eight repeat units for mono SSRs, four repeat units for di- and trinucleotide repeat SSRs and three repeat units for tetra-, penta- and hexanucleotide repeat SSRs.

## 5. Conclusions

In conclusion, whole-plastid-genome data have been established as a powerful tool to understand evolutionary trends in Asteraceae, adding support to previous systematic inferences based on different markers. Our results confirm the double inversion in plastid DNA occurring during the early evolution of Asteraceae and reveal an additional structural change—appearance of a *rps*19 pseudogene—that could be evolutionarily linked to the two inversions. Our work also contributed information about the gene composition, nucleotide diversity and repeat content in Asteraceae plastomes, which could be useful for the design of novel molecular markers for phylogeographic and population genetic studies. Finally, the phylogenomic reconstruction based on whole-plastome data clarified previous uncertain questions on Asteraceae systematics at the tribe level while also exposing some major incongruences among the evolutionary histories revealed by nuclear and plastid DNA data.

## Figures and Tables

**Table 1:** Species information, SRA and GenBank accessions and mapping details for Asteraceae and the outgroup.

Species	Family/Tribe	SRA/GenBank	Min	Max	X	SD	Ns	Ns (%)	Genome Size (bp)	GC (%)	LSC Length (bp)	SSC Length (bp)	IR_A-B_ Length (bp)
*Achillea millefolium*	Anthemideae	SRR17032110	10	176	92.9	29.5	5519	3.70	149,113	37.9	82,450	18,406	24,126–24,131
*Anacyclus radiatus*	Anthemideae	SRR9822607	10	96	54	10.2	1247	0.83	149,866	37.5	82,481	18,427	24,479–24,479
*Argyranthemum foeniculaceum*	Anthemideae	SRR17032109	10	235	124.2	37.5	5698	3.80	149,841	37.9	82,471	18,396	24,487–24,487
*Artemisia tridentata*	Anthemideae	SRR17032104	10	248	11.4	11.7	10	0.01	151,143	37.4	82,916	18,295	24,966–24,966
*Santolina chamaecyparissus*	Anthemideae	SRR17032107	10	170	760	25	91	0.06	149,733	37.4	82,463	18,304	24,483–24,483
*Tanacetum cinerariifolium*	Anthemideae	DRR180629	36	281	183.6	30.4	0	0.00	150,139	37.4	82,723	18,442	24,487–24,487
*Centipeda minima*	Anthemideae	SRR8666707	10	154	83.1	15.7	3	0.00	152,432	37.5	84,125	18,367	24,970–24,970
*Aster tataricus*	Astereae	NC042913	GenBank						152,992	37.3	84,702	18,244	25,023–25,023
*Conyza bonariensis*	Astereae	MF276802	GenBank						153,014	37.2	84,655	18,358	24,998–25,003
*Bahia ambrosioides*	Bahieae	SRR17032103	10	424	252.1	62	845	0.56	151,377	37.6	83,539	17,866	25,028–24,944
*Fulcaldea stuessyi*	Barnadesieae	SRR2154060	10	234	123	33.1	2	0.00	152,890	37.8	83,894	18,664	25,191–25,141
*Archidasphyllum excelsum*	Barnadesieae	MH298332	GenBank						151,880	37.8	83,219	185,94	25,030–25,037
*Doniophyton anomalum*	Barnadesieae	MH899017	GenBank						150,547	38.0	82,297	18,785	24,743–24,722
*Calendula arvensis*	Calenduleae	SRR17032101	10	706.5	1630	155.8	1062	0.71	150,451	37.7	83,118	17,859	24,737–24,737
*Lactuca sativa*	Cichorieae	NC007578	GenBank						152,765	37.5	84,103	18,502	25,032–25,128
*Sonchus oleraceus*	Cichorieae	SRR8666672	110	577	424.1	61.5	0	0.00	151,807	37.6	84,170	18,115	24,717–24,805
*Tragopogon porrifolius*	Cichorieae	SRS10264650	81	2422	1441.7	167.9	0	0.00	153,047	37.7	84,292	18,347	25,245–25,163
*Bidens subalternans*	Coreopsideae	SRR17032102	10	688	392.3	79.4	287	0.19	151,433	37.5	83,947	18,151	24,678–24,657
*Coreopsis gigantea*	Coreopsideae	SRR17032100	10	811	329.4	91.7	1381	0.91	151,745	37.5	83,776	18,131	24,925–24,913
*Carthamus tinctorius*	Cardueae	NC030783	GenBank						153,205	37.8	94,217	18,610	25,189–25,189
*Ageratum houstonianum*	Eupatorieae	SRR7121578	199	541	373.7	49.5	0	0.00	151,541	37.4	83,367	18,388	24,893–24,893
*Eupatorium cannabinum*	Eupatorieae	SRR17032099	10	442	315	52.2	0	0.00	151,384	37.6	83,102	18,316	24,983–24,983
*Helichrysum splendidum*	Gnaphalieae	SRR17032098	14	350	184.4	42.6	0	0.00	153,491	37.0	85,228	18,525	24,839–24,839
*Phagnalon saxatile*	Gnaphalieae	SRR17032108	30	383	242.6	52.5	0	0.00	152,680	37.5	84,180	18,495	25,013–24,992
*Arnica montana*	Madieae	SRR17032105	10	274	127.7	28.9	2124	1.40	151,998	37.6	83,839	18,309	24,925–24,925
*Helianthus annuus*	Heliantheae	NC007977	GenBank						151,104	37.6	83,612	18,326	24,583–24,583
*Pterocaulon sphaeranthoides*	Inuleae	SRR8666812	10	124	65.7	15	1	0.00	152,219	37.6	84,069	18,168	24,991–24,991
*Streptoglossa adscendens*	Inuleae	SRR8666220	10	172	79.7	22.1	18	0.01	152,290	37.5	84,126	18,176	24,994–24,994
*Melampodium linerarilobum*	Millerieae	ERR3909555	10	408	179.6	30.8	77	0.05	153,872	37.6	85,083	18,786	25,035–24,968
*Sigesbeckia orientalis*	Millerieae	SRR8666701	38	211	131.7	22.5	0	0.00	151,797	37.6	83,624	18,215	24,979–24,979
*Gerbera hybrida*	Mutisieae	SRR2154064	10	1296	513.1	147.3	4	0.00	154,946	37.7	83,462	18,217	25,151–25,116
*Senecio vulgaris*	Senecioneae	SRR2155042	88	1040	646.6	158.9	0	0.00	150,802	37.3	82,890	18,212	24,818–24,882
*Tussilago farfara*	Senecioneae	SRR17032106	10	606	265.2	83.6	4986	3.32	150,314	37.2	82,503	18,187	24,800–24,824
*Flaveria trinervia*	Tageteae	SRR8666717	10	80	39.1	9.5	3	0.00	152,410	37.6	83,997	18,419	24,997–24,997
*Acilepis saligna*	Vernonieae	SRR7121903	10	296	207	22.9	0	0.00	152,918	37.7	84,093	18,756	25,088–24,981
*Pleurocarpaea gracilis*	Vernonieae	SRR8666739	10	77	40.6	8.4	122	0.08	152,432	37.7	83,569	18,531	25,172–25,160
*Nastanthus patagonicus*	Fam. Calyceraceae	SRR2153911	124	6021	2326.8	1175.8	0	0.00	152,554	38.1	83,772	18,658	25,119–25,005

**Table 2:** Collection date and geographic locality of the gathered plant materials.

Species	Collection Date	Geographic Locality
*Achillea millefolium*	06 October 2018	Catalonia, Girona, Llinars
*Argyranthemum foeniculaceum*	27 September 2019	Catalonia, Barcelona, Botanical Garden of Barcelona
*Arnica montana*	23 September 2018	Catalonia, Barcelona, Botanical Garden of Barcelona
*Artemisia tridentata*	27 September 2019	Catalonia, Barcelona, Botanical Garden of Barcelona
*Bahia ambrosioides*	27 September 2019	Catalonia, Barcelona, Botanical Garden of Barcelona
*Bidens subalternans*	13 September 2018	Catalonia, Barcelona, Caldes de Montbui
*Calendula arvensis*	06 October 2018	Catalonia, Barcelona, Caldes de Montbui
*Coreopsis gigantea*	27 September 2019	Catalonia, Barcelona, Botanical Garden of Barcelona
*Eupatorium cannabinum*	23 September 2018	Catalonia, Girona, Setcases
*Helichrysum splendidum*	27 September 2019	Catalonia, Barcelona, Botanical Garden of Barcelona
*Phagnalon saxatile*	27 September 2019	Catalonia, Barcelona, Botanical Garden of Barcelona
*Santolina chamaecyparissus*	27 September 2019	Catalonia, Barcelona, Botanical Garden of Barcelona
*Tussilago farfara*	23 September 2018	Catalonia, Girona, Setcases

## Data Availability

Data is available at the National Center for Biotechnology Information (NCBI) library under the accession PRJNA764566 titled “Deconstructing ribosomal DNA: from the sequence to the chromosome across the Tree-of-Life (rDNA-LIFE)”.

## Supplementary Materials

The following are available online at https://www.mdpi.com/article/10.3390/plants10122699/s1, Figure S1: Phylogeny reconstructed using Bayesian inference (BI) of family Asteraceae, representing the relationship among 18 tribes, excluding the subfamily Barnadesioideae and the outgroup *Nastanthus patagonicus* (family Calyceraceae). Three subsets were used: (A) coding DNA sequences, (B) coding DNA sequences, transfer RNAs and ribosomal RNAs, (C) the whole plastid sequences, Figure S2: Mauve alignment of 37 plastid genomes of species from families Asteraceae and Calyceraceae, sorted phylogenetically. Within each group, local collinear blocks are represented by blocks of the same color and connected by lines. The hypothetical origin of the rearrangement is represented by a star in the phylogenetic tree, Figure S3: Comparison of the boundaries of the LSC, SSC and IR regions of a subset of 37 species from families Asteraceae and Calyceraceae. Genes suffixed with a phi (φ) are potential pseudogenes, Figure S4: Number of different long-repeat sequence types found in the plastid genomes of the families Asteraceae and Calyceraceae, Figure S5: Number of SSR loci analyzed and number of tandem repeats identified for each species of the families Asteraceae and Calyceraceae, Table S1: List of genes annotated in the studied plastid genomes of 37 species of Asteraceae and Calyceraceae, Table S2: Models used for phylogenetic analyses, Table S3: Microsatellite distribution, frequency and number for each species of this study.

## References

[1] Funk V.A., Anderberg A.A., Baldwin B.G., Bayer R.J., Bonifacino J.M., Breitwieser I., Brouillet L., Carbajal R., Chan R., Coutinho A.X.P., Funk V.A., Susanna A., Stuessy T.F., Bayer R.J. (2009). Compositae metatrees: The next generation. Systematics, Evolution and Biogeography of Compositae.

[2] Kadereit J.W., Jeffrey C., Kubitzki K. (2007). Flowering plants. Eudicots Asterales. The Families and Genera of Vascular Plants.

[3] Stuessy T.F., Spooner D.M., Evans K.A. (1986). Adaptive significance of ray corollas in *Helianthus grosseserratus* (Compositae). Am. Midl. Nat..

[4] Anderberg A.A., Baldwin B.G., Bayer R.G., Breitwieser J., Jeffrey C., Dillon M.O., Eldenäs P., Funk V., Garcia-Jacas N., Hind D.J.N., Kubitzki K., Kadereit J.W., Jeffrey C. (2007). Compositae. Flowering Plants, Eudicots: Asterales. The Families and Genera of Vascular Plants.

[5] Semple J.C., Watanabe K., Funk V.A., Susanna A., Stuessy T.F., Bayer R.J. (2009). A review of chromosome numbers in Asteraceae with hypotheses on chromosomal base number evolution. Systematics, Evolution and Biogeography of Compositae.

[6] Barker M.S., Li Z., Kiddler T.I., Reardon C.R., Lai Z., Oliveira L.O., Scacitelli M., Rieseberg L.H. (2016). Most Compositae (Asteraceae) are descendants of a paleohexaploid and all share a paleotetraploid ancestor with the Calyceraceae. Am. J. Bot..

[7] Barber J.C., Finch C.C., Francisco-Ortega J., Santos-Guerra A., Jansen R.K. (2007). Hybridization in Macaronesian *Sideritis* (Lamiaceae): Evidence from incongruence of multiple independent nuclear and chloroplast sequence datasets. Taxon.

[8] Jones K.E., Reyes-Betancort J.A., Hiscock S.J., Carine M.A. (2014). Allopatric diversification, multiple habitat shifts, and hybridization in the evolution of *Pericallis* (Asteraceae), A Macaronesian endemic genus. Am. J. Bot..

[9] Garcia S., Panero J.L., Siroky J., Kovarik A. (2010). Repeated reunions and splits feature the highly dynamic evolution of 5S and 35S ribosomal RNA genes (rDNA) in the Asteraceae family. BMC Plant Biol..

[10] Panero J.L., Francisco-Ortega J., Jansen R.K., Santos-Guerra A. (1999). Molecular evidence for multiple origins of woodiness and a New World biogeographic connection of the Macaronesian Island endemic *Pericalis* (Asteraceae: Senecioneae). Proc. Natl. Acad. Sci. USA.

[11] Jabaily R.S., Shepherd K.A., Gardner A.G., Gustafsson M.H., Howarth D.G., Motley T.J. (2014). Historical biogeography of the predominantly Australian plant family Goodeniaceae. J. Biogeogr..

[12] Denham S.S., Zavala-Gallo L., Johnson L.A., Pozner R.E. (2016). Insights into the phylogeny and evolutionary history of Calyceraceae. Taxon.

[13] Kim K.J., Jansen R.K., Wallace R.S., Michaels H.J., Palmer J.D. (1992). Phylogenetic implications of *rbc*L sequence variation in the Asteraceae. Ann. Mo. Bot. Gard..

[14] Kim K.J., Choi K.S., Jansen R.K. (2005). Two chloroplast DNA inversion originated simultaneously during the early evolution of the sunflower family (Asteraceae). Mol. Biol. Evol..

[15] Shaw J., Lickey E.B., Schilling E.E., Small R.L. (2007). Comparison of whole chloroplast genome sequences to choose noncoding regions for phylogenetic studies in angiosperms: The tortoise and the hare III. Am. J. Bot..

[16] Panero J.L., Crozier B.S. (2016). Macroevolutionary dynamics in the early diversification of Asteraceae. Mol. Phylogenetics Evol..

[17] Mandel J.R., Dikow R.B., Siniscalchi C.M., Thapa R., Watson L.E., Funk V.A. (2019). A fully resolved backbone phylogeny reveals numerous dispersals and explosive diversifications throughout the history of Asteraceae. Proc. Natl. Acad. Sci. USA.

[18] Mower J.P., Vickrey T.L., Chaw S.M., Jansen R.K. (2018). Structural diversity among plastid genomes of land plants. Advances in Botanical Research.

[19] Loeuille B., Thode V., Siniscalchi C., Andrade S., Rossi M., Pirani J.R. (2021). Extremely low nucleotide diversity among thirty-six new chloroplast genome sequences from *Aldama* (Heliantheae, Asteraceae) and comparative chloroplast genomics analyses with closely related genera. PeerJ.

[20] Ruhlman T.A., Jansen R.K., Chaw S.M., Jansen R.K. (2018). Aberration or analogy? The atypical plastomes of Geraniaceae. Advances in Botanical Research.

[21] Lee Y.S., Park J.Y., Kim J.K., Lee H.O., Park H.S., Lee S.C., Kang J.H., Lee T.J., Hung S.H., Yang T.J. (2021). The complete chloroplast genome sequences of *Artemisia gmelinii* and *Artemisia capillaris* (Asteraceae). Mitochondrial DNA Part B.

[22] Dierckxsens N., Mardulyn P., Smits G. (2017). NOVOPlasty: De novo assembly of organelle genomes from whole genome data. Nucleic Acids Res..

[23] Luo R., Liu B., Xie Y., Huang W., Yuan J., He G., Chen Y., Pan Q., Liu Y., Tang J. (2012). SOAPdenovo2: An empirically improved memory-efficient short-read *de novo* assembler. Gigascience.

[24] Robinson H. (2004). New supertribes Helianthodae and Senecionodae, for the subfamily Asteroideae (Asteraceae). Phytologia.

[25] Xiao-Ming Z., Junrui W., Li F., Sha L., Hongbo P., Lan Q., Jing L., Yan S., Weihua Q., Lifang Z. (2017). Inferring the evolutionary mechanism of the chloroplast genome size by comparing whole-chloroplast genome sequences in seed plants. Sci. Rep.-UK.

[26] Palmer J.D., Stein D.B. (1986). Conservation of chloroplast genome structure among vascular plants. Curr. Genet..

[27] Wang W., Lanfear R. (2019). Long-reads reveal that the chloroplast genome exists in two distinct versions in most plants. Genome Biol. Evol..

[28] Walker J.F., Zanis M.J., Emery N.C. (2014). Comparative analysis of complete chloroplast genome sequence and inversion variation in *Lasthenia burkei* (Madieae, Asteraceae). Am. J. Bot..

[29] Wang M., Cui L., Feng K., Deng P., Du X., Wan F., Weining S., Nie X. (2015). Comparative analysis of Asteraceae chloroplast genomes: Structural organization, RNA editing and evolution. Plant Mol. Biol. Rep..

[30] Jansen R.K., Palmer J.D. (1987). A chloroplast DNA inversion marks an ancient evolutionary split in the sunflower family (Asteraceae). Proc. Natl. Acad. Sci. USA.

[31] Wang R.J., Cheng C.L., Chang C.C., Wu C.L., Su T.M., Chaw S.M. (2008). Dynamics and evolution of the inverted repeat-large single copy junctions in the chloroplast genome of monocots. BMC Evol. Biol..

[32] Ni L., Zhao Z., Xu H., Chen S., Dorje G. (2017). Chloroplast genome structures in *Gentiana* (Gentianaceae), based on three medicinal alpine plants used in Tibetan herbal medicine. Curr. Genet..

[33] Ma Q., Li S., Bi C., Hao Z., Sun C., Ye N. (2017). Complete chloroplast genome sequence of a major economic species, *Ziziphus jujuba* (Rhamnaceae). Curr. Genet..

[34] Shen X., Wu M., Liao B., Liu Z., Bai R., Xiao S., Li X., Zhang B., Xu J., Chen S. (2017). Complete chloroplast genome sequence and phylogenetic analysis of the medicinal plant *Artemisia annua*. Molecules.

[35] Nevill P.G., Zhong X., Tonti-Filippini J., Byrne M., Hislop M., Thiele K., van Leeuwen S., Boykin L.M., Small I. (2020). Large scale genome skimming from herbarium material for accurate plant identification and phylogenomics. BMC Plant Methods.

[36] Belser C., Istace B., Denis E., Dubarry M., Baurens F.C., Falentin C., Genete M., Berrabah W., Chèvre A.M., Delourme R. (2018). Chromosome-scale assemblies of plant genomes using nanopore long reads and optical maps. Nat. Plants.

[37] Small R.L., Ryburn J.A., Cronn R.C., Seelanan T., Wendel J.F. (1998). The tortoise and the hare: Choosing between noncoding plastome and nuclear *Adh* sequences for phylogeny reconstruction in a recently diverged plant group. Am. J. Bot..

[38] Asaf S., Khan A.L., Khan A.R., Waqas M., Kang S.M., Khan M.A., Lee S.M., Lee I.J. (2016). Complete chloroplast genome of *Nicotiana otophora* and its comparison with related species. Front. Plant Sci..

[39] Gichira A.W., Avoga S., Li Z., Hu G., Wang Q., Chen J. (2019). Comparative genomics of 11 complete chloroplast genomes of Senecioneae (Asteraceae) species: DNA barcodes and phylogenetics. Bot. Stud..

[40] Wang H., Liu X., Moore M.J., Landrein S., Liu B., Zhu Z.X., Wang H.F. (2020). Plastic phylogenomic insights into the evolution of the Caprifoliaceae sl (Dipsacales). Mol. Phylogenetics Evol..

[41] Fang J., Lin A., Yuan X., Chen Y., He W., Huang J., Zhang X., Lin G., Zhang J., Xue T. (2020). The complete chloroplast genome of *Isochrysis galbana* and comparison with related haptophyte species. Algal Res..

[42] Koch M., Haubold B., Mitchell-Olds T. (2001). Molecular systematics of the Brassicaceae: Evidence from coding plastidic *mat*K and nuclear *Chs* sequences. Am. J. Bot..

[43] Bogler D.J., Pires J.C., Francisco-Ortega J. (2006). Phylogeny of Agavaceae based on *ndh*F, *rbc*L and ITS sequences. Aliso A J. Syst. Evol. Bot..

[44] Milligan B.G., Hampton J.N., Palmer J.D. (1989). Dispersed repeats and structural reorganization in subclover chloroplast DNA. Mol. Biol. Evol..

[45] Sun J., Wang Y., Liu Y., Xu C., Yuan Q., Guo L., Huang L. (2020). Evolutionary and phylogenetic aspects of the chloroplast genome of *Chaenomeles* species. Sci. Rep.-UK.

[46] Xu D., Abe J., Gai J., Shimamoto Y. (2002). Diversity of chloroplast DNA SSRs in wild and cultivated soybeans: Evidence for multiple origins of cultivated soybean. Theor. Appl. Genet..

[47] Mariotti R., Cultrera N.G., Díez C.M., Baldoni L., Rubini A. (2010). Identification of new polymorphic regions and differentiation of cultivated olives (*Olea europaea* L.) through plastome sequence comparison. BMC Plant Biol..

[48] Yang J.Y., Pak J.H., Kim S.C. (2018). The complete plastome sequence of *Rubus takesimensis* endemic to Ulleung Island, Korea: Insights into molecular evolution of anagenetically derived species in *Rubus* (Rosaceae). Gene.

[49] Zhang C., Huang C.H., Liu M., Hu Y., Panero J.L., Luebert F., Gao T., Ma H. (2021). Phylotranscriptomic insights into Asteraceae diversity, polyploidy, and morphological innovation. J. Integr. Plant Biol..

[50] Gruenstaeudl M., Jenke N. (2020). PACVr: Plastome assembly coverage visualitzation in R. BMC Bioinform..

[51] Shen X., Guo S., Yin Y., Zhang J., Yin X., Liang C., Wang Z., Huang B., Liu Y., Xiao S. (2018). Complete chloroplast genome sequence and phylogenetic analysis of *Aster tataricus*. Molecules.

[52] Hereward J.P., Werth J.A., Thornby D.F., Keenan M., Chauhan B.S., Walter G.H. (2017). Complete chloroplast genome of glyphosate resistant *Conyza bonariensis* (L.) Cronquist from Australia. Mitochondrial DNA Part B.

[53] Timme R.E., Kuehl J.V., Boore J.L. (2007). A comparative analysis of the *Lactuca* and *Helianthus* (Asteraceae) plastid genomes: Identification of divergent regions and categorization of shared repeats. Am. J. Bot..

[54] Kanamoto H., Yamashita A., Okumura S., Hattori M., Tomizawa K.I. (2004). The complete genome sequence of the *Lactuca sativa* (lettuce) chloroplast. Plant Cell Physiol..

[55] Doyle I.J., Doyle J.L. (1987). A Rapid DNA Isolation Procedure for Small Quantities of Fresh Leaf Tissue. Phytochem. Bull..

[56] Tillich M., Lehwark P., Pellizzer T., Ulbricht-Jones E.S., Fischer A., Bock R., Greiner S. (2017). GeSeq-versatile and accurate annotation of organelle genomes. Nucleic Acids Res..

[57] Katoh K., Standley D.M. (2013). MAFFT multiple sequence alignment software version 7: Improvements in performance and usability. Mol. Biol. Evol..

[58] Ronquist F., Teslenko M., Van Der Mark P., Ayres D.L., Darling A., Höhna S., Larget B., Liu L., Suchard M.A., Huelsenbeck J.P. (2012). MrBayes 3.2: Efficient bayesian phylogenetic inference and model choice across a large model space. Syst. Biol..

[59] Miller M.A., Pfeiffer W., Schwartz T. (2010). Creating the CIPRES science gateway for inference of large phylogenetic trees. Gatew. Comput. Environ. Work GCE.

[60] Stamatakis A. (2006). RAxML-VI-HPC: Maximum likelihood-based phylogenetic analyses with thousands of taxa and mixed models. Bioinformatics.

[61] Lanfear R., Frandsen P.B., Wright A.M., Senfeld T., Calcott B. (2017). PartitionFinder 2: New methods for selecting partitioned models of evolution for molecular and morphological phylogenetic analyses. Mol. Biol. Evol..

[62] Darling A.C., Mau B., Blattner F.R., Perna N.T. (2004). MAUVE: Multiple alignment of conserved genomic sequence with rearrangements. Genome Res..

[63] Rozas J., Ferrer-Mata A., Sánchez-DelBarrio J.C., Guirao-Rico S., Librado P., Ramos-Onsins S.E., Sánchez-Gracia A. (2017). DNASP 6: DNA sequence polymorphism analysis of large datasets. Mol. Biol. Evol..

[64] Kurtz S., Choudhuri J.V., Ohlebusch E., Schleiermacher C., Stoye J., Giegerich R. (2001). REPuter: The manifold applications of repeat analysis on a genomic scale. Nucleic Acids Res..

[65] Sun M., Soltis D.E., Soltis P.S., Zhu X., Burleigh J.G., Chen Z. (2015). Deep phylogenetic incongruence in the angiosperms clade Rosidae. Mol. Phylogenetics Evol..

[66] Benson G. (1999). Tandem Repeat Finder: A program to analyze DNA sequences. Nucleic Acids Res..

[67] Beier S., Thiel T., Münch T., Scholz U., Mascher M. (2017). MISA-web: A web server for microsatellite prediction. Bioinformatics.

