# Supplementary material for: Plastome Diversity and Phylogenomic Relationships in Asteraceae
Source: Plants (Basel). 2021 Dec 8;10(12):2699. doi: 10.3390/plants10122699 (PMC8705268; doi:10.3390/plants10122699)
Supplement: Supplementary file 1 [file plants-10-02699-s001.zip › suppl/table S1, table S2.pdf]

Table S1. List of genes annotated in the studied plastid genomes of 37 species of Asteraceae and Calyceraceae.

Gene family	Gene
Transfer RNA	*trn*A-UGC, *trn*C-GCA, *trn*D-GUC, *trn*E-UUC, *trn*F-GAA, *trnf*M-CAU, *trn*G-GCC, *trn*G-UCC, *trn*H-GUG, *trn*I-CAU, *trnI*-GAU, *trn*K-UUU, *trn*L-CAA, *trn*L-UAA, *trn*L-UAG, *trn*M-CAU, *trn*N-GUU, *trn*P-UGG, *trn*Q-UGG, *trn*R-ACG, *trn*R-UCU, *trn*S-GCU, *trn*S-GGA, *trn*S-UGA, *trn*T-GGU, *trn*T-UGU, *trn*V-GAC, *trn*V-UAC, *trn*W-CCA, *trn*Y-GUA
Small ribosomal units	*rps*2, *rps*3, *rps*4, *rps*7, *rps*8, *rps*11, *rps*12, *rps*14, *rps*15, *rps*16, *rps*18, *rps*19
Large ribosomal units	*rpl*2, *rpl*14, *rpl*16, *rpl*20, *rpl*22, *rpl*23, *rpl*32, *rpl*33, *rpl*36
RNA polymerase sub-units	*rpo*A, *rpo*B, *rpo*C1, *rpo*C2
Translation initiation facto	*inf*A
NADH dehydrogenase	*ndh*A, *ndh*B, *ndh*C, *ndh*D, *ndh*E, *ndh*F, *ndh*G, *ndh*H, *ndh*I, *ndh*J, *ndh*K
Photosystem I	*pbf*1, *psa*A, *psa*B, *psa*C, *psa*I, *psa*J, *ycf*3, *ycf*4
Photosystem II	*psb*A, *psb*B, *psb*C, *psb*D, *psb*E, *psb*F, *psb*H, *psb*I, *psb*J, *psb*K, *psb*L, *psb*M, *psb*T, *psb*Z
Cytochrome b/f complex	*pet*A, *pet*B, *pet*D, *pet*G, *pet*L, *pet*N
ATP synthase	*atp*A, *atp*B, *atp*E, *atp*F, *atp*H, *atp*I, *ycf*2
Large subunit of rubisco	*rbc*L
Maturase	*mat*K
Protease	*clp*P
Acetyl-CoA-carboxylase sub-unit	*acc*D
Envelope membrane protein	*cem*A
Component of TIC complex	*Ycf*1
c-Type cytochrome synthesis	*ccs*A
Uncharacterized protein	*ycf*15
Ribosomal RNA	*rrn*4.5, *rrn*5, *rrn*16, *rrn*23

Table S2. Models used for phylogenetic analyses.

**Inference Method**	**Coding regions (CDS)**			**tRNA**	**rRNA**
	Codon 1	Codon 2	Codon 3		
**Maximum Likelihood**	GTR + γ	GTR + γ	GTR + γ	GTR + γ	GTR + γ
**Bayesian Inference**	GTR + inv_γ	GTR + inv_γ	GTR + inv_γ	GTR + inv_γ	K80 + inv_γ
