# Supplementary figures and images for: Plastome Diversity and Phylogenomic Relationships in Asteraceae
Source: Plants (Basel). 2021 Dec 8;10(12):2699. doi: 10.3390/plants10122699 (PMC8705268; doi:10.3390/plants10122699)

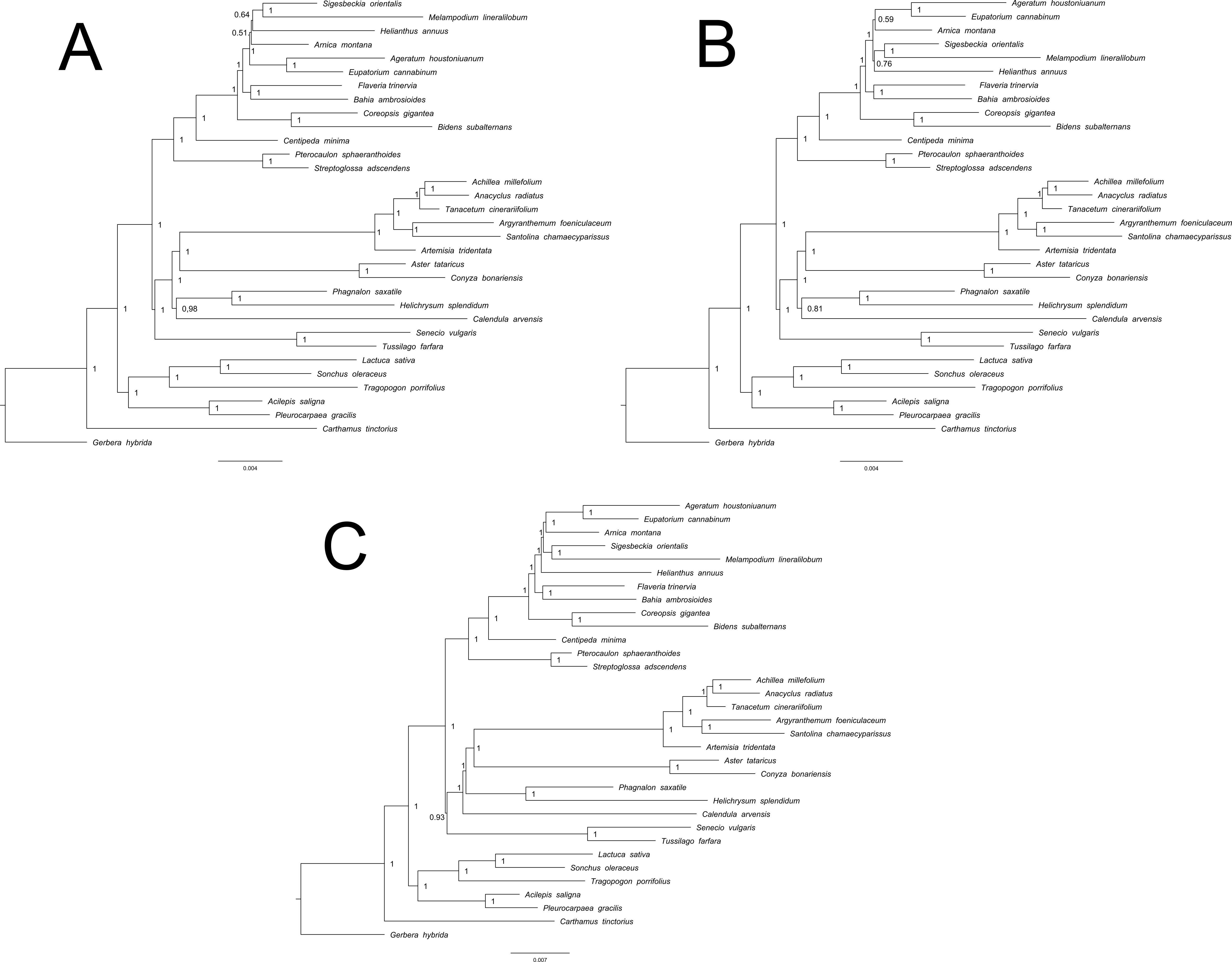

Supplement: Supplementary file 1 [file plants-10-02699-s001.zip › suppl/Figure_S1.png]

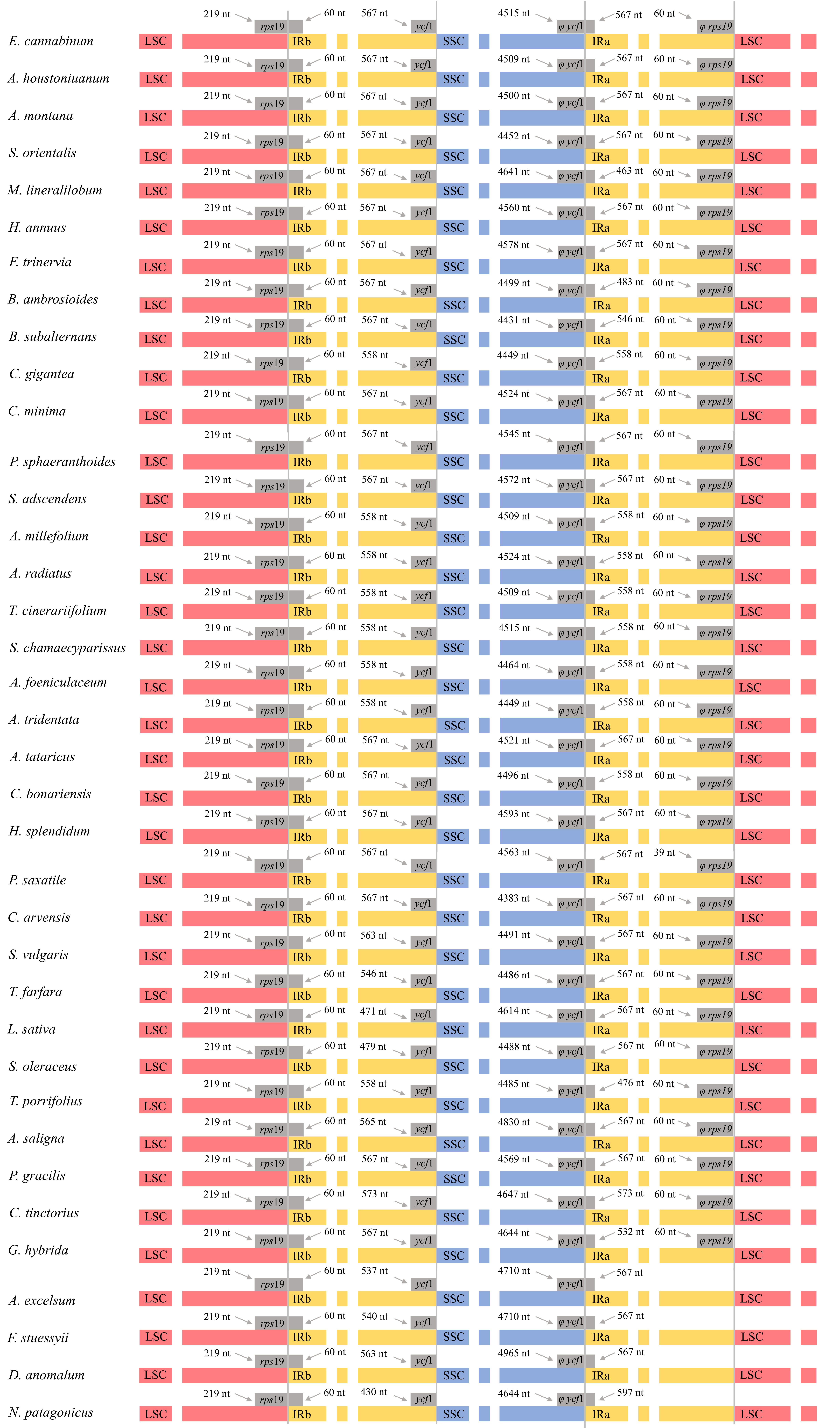

Supplement: Supplementary file 1 [file plants-10-02699-s001.zip › suppl/Figure_S3.png]

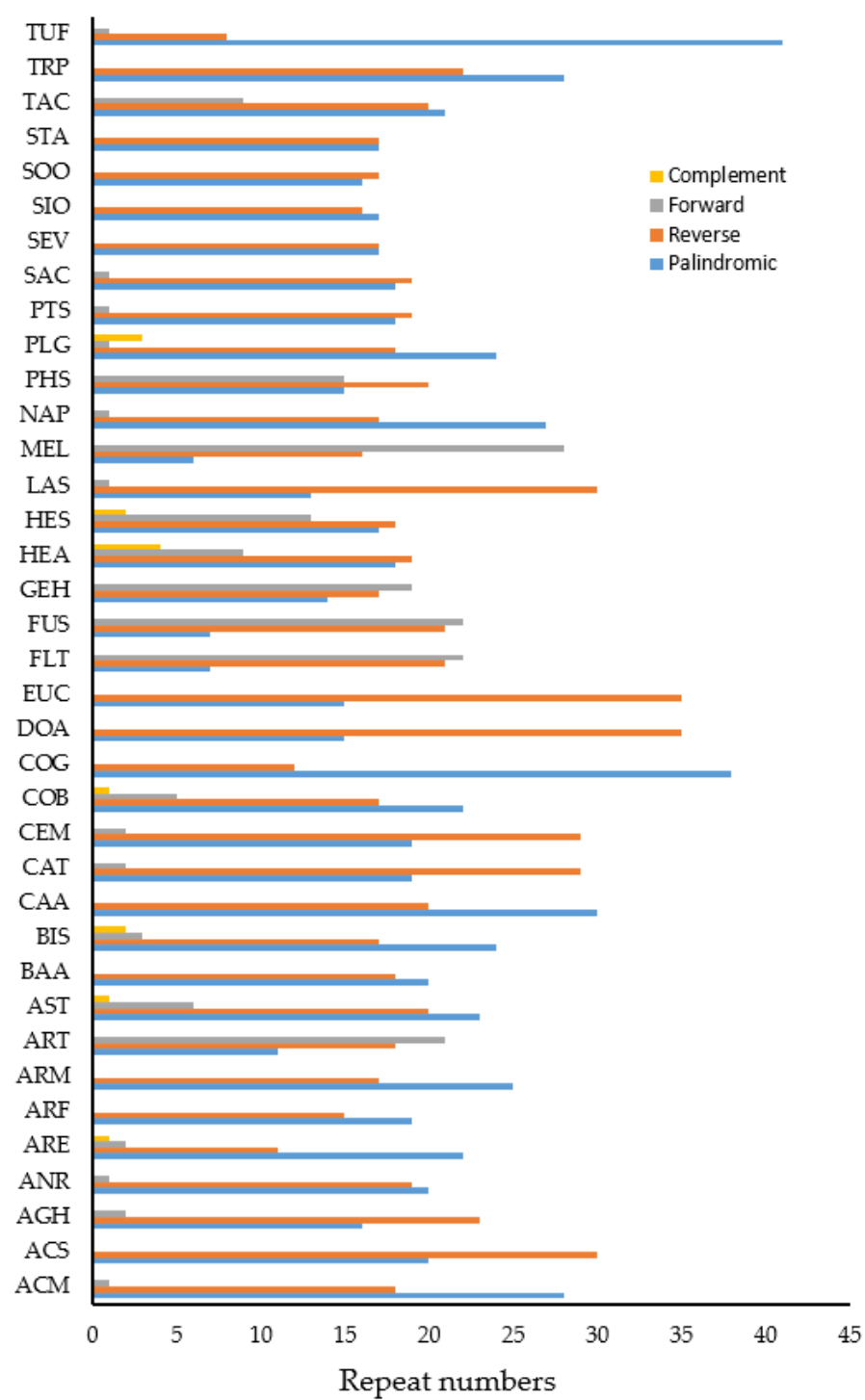

Supplement: Supplementary file 1 [file plants-10-02699-s001.zip › suppl/Figure_S4.pdf]

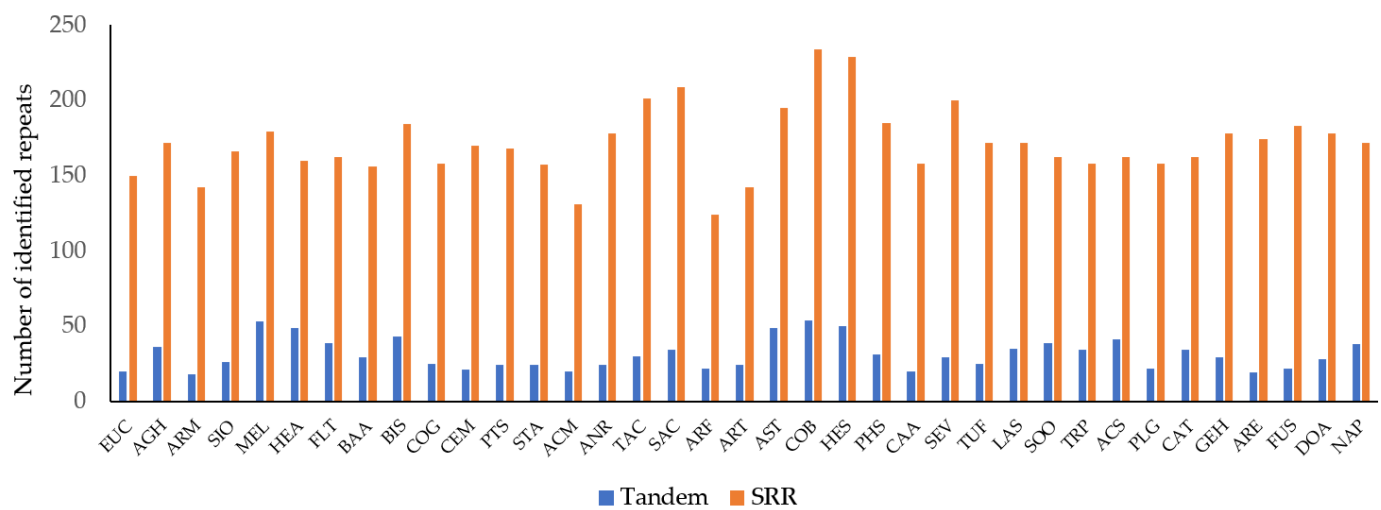

Supplement: Supplementary file 1 [file plants-10-02699-s001.zip › suppl/Figure_S5.pdf]
